# Prognostic significance of cancer stemness‐associated genes in patients with gliomas

**DOI:** 10.1002/ctm2.186

**Published:** 2020-09-27

**Authors:** Shanqiang Qu, Jing Huang, Jin Liu, Huafu Wang

**Affiliations:** ^1^ Department of Neurosurgery Lishui People's Hospital (The sixth affiliated Hospital of Wenzhou Medical University) Lishui P. R. China; ^2^ Department of Neurosurgery The First Affiliated Hospital of Sun Yat‐sen University Guangzhou P. R. China; ^3^ Department of Stomatology The Seventh Affiliated Hospital of Sun Yat‐sen University Shenzhen P. R. China; ^4^ Department of Clinical Pharmacy Lishui People's Hospital (The sixth affiliated Hospital of Wenzhou Medical University) Lishui P. R. China

**Dear Editor,**

Glioma is the most common type of malignant intracranial tumor, with glioma morbidity accounting for half of that of all brain tumours.^1^ Although most patients with gliomas are routinely offered radiotherapy and temozolomide chemotherapy after tumor resection, the prognosis is still very poor. Patients surviving more than 5 years account for less than 5% of all patients.^2^ Increasing studies have indicated that cancer stem cells (CSCs) play a key role in tumorigenesis, progression, and drug resistance^3^, ^4^; therefore, our study aimed to identify novel prognostic molecules and construct a prognostic model that integrates clinical features and cancer stemness‐associated genes for use in glioma patients.

First, data from 301 patients with WHOII‐IV gliomas (dataset ID:mRNAarray, data type: mRNA Microarray) were extracted from the Chinese Glioma Genome Atlas (CGGA) database (http://cgga.org.cn/download.jsp). The mean age of the patients was 42.4 years (range 12‐70 years). The baseline characteristics of the patients are summarized in Supporting information Table S2. Then, to identify the prognostic biomarkers of patients, 359 cancer stemness‐associated genes (relevance score ≥ 50 points) screened from the GeneCards database were included in the univariate Cox regression analysis, from which 162 genes (*P *≤ .001) were identified. Next, these significant genes were further entered into multivariate analysis, and we found that 12 statistically significant genes were independent prognostic factors (Supporting information Table S1). The description, molecular function, and regression coefficient of these genes are summarized in Supporting information Table S3.

Next, the stemness score of each patient was calculated according to the mRNA levels and regression coefficient of each prognostic gene. According to the median cut‐off of the stemness score, 301 glioma patients were divided into low (n = 150) and high stemness score groups (n = 151). We used χ^2^ tests to investigate the correlation between the stemness score and clinicopathological features and found that the stemness score was significantly correlated with age (*P *= .002), WHO grade (*P *< .001), histopathology (*P *< .001), IDH mutation (*P *< .001), and 1p/19q codeletion (*P *= .001) (Table 1). Moreover, Figure 1A‐F shows the distribution of stemness scores in the different subgroups based on age, sex, WHO grade, IDH status, 1p/19q status, and recurrence (*P *< .001, *P *= .44, *P *< .0001, *P *< .0001, *P *< .0001 and *P *= .11, respectively). We additionally analyzed the association between the stemness score and common biomarkers, including Ki‐67, vimentin, and CDH1 (E‐cadherin). Surprisingly, the scatter plots showed that the stemness score was positively related to the mRNA levels of Ki‐67 and vimentin (*r* = .35, *P *< .0001; *r* = .65, *P *< .0001, respectively) but negatively related to CDH1 mRNA levels (*r* = −0.12, *P *< .05) (Figure 1G‐I).

**TABLE 1:** The correlation between the stemness score and clinical features

	Stemness score		
Characteristics	Low score	High score	*P*‐value
Age			
≥40	69	96	.002
<40	80	54	
Gender			
Male	88	92	.689
Female	62	59	
WHO grade			
WHOII	96	21	<.001
WHOIII	21	36	
WHOIV	32	92	
Histopathology			
O	17	1	<.001
OA	34	2	
A	45	18	
AO	7	7	
AOA	0	17	
AA	4	12	
GBM	32	92	
IDH			
Mutation	8	36	<.001
Wildtype	51	114	
1p/19q			
Codel	15	1	.001
Non‐codel	35	41	
Radiotherapy			
Yes	123	126	.710
No	20	18	
Chemotherapy			
Yes	66	85	.067
No	69	57	
Recurrence			
Yes	12	20	.274
No	135	129	

A, astrocytoma; AA, anaplastic astrocytoma; AO, anaplastic oligodendroglioma; AOA, anaplastic oligoastrocytoma; GBM, glioblastoma, O, oligodendroglioma; OA, oligoastrocytoma.

**FIGURE 1:**
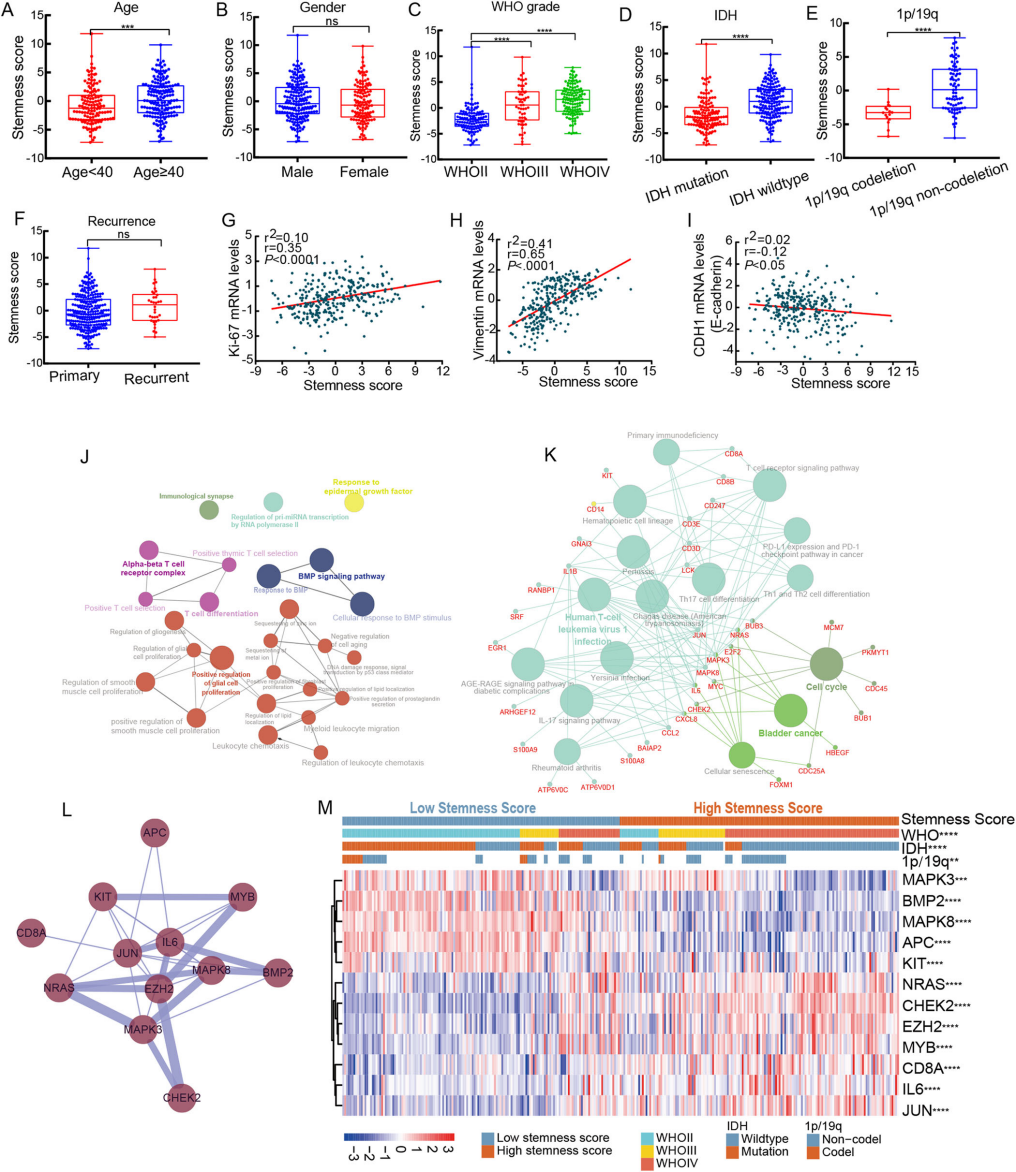
Correlation between stemness score and common clinicopathological features, functional analyses of cancer stemness‐associated genes. The distribution of stemness score in glioma patients grouped by age (A), gender (B), WHO grade (C), IDH status (D), 1p/19q status (E), and tumor recurrence (F). (G) Scatter plot between stemness score and ki‐67 mRNA levels. (H) Scatter plot between stemness score and vimentin mRNA levels. (I) Scatter plot between stemness score and CDH1 mRNA levels. (J) GO and (K) KEGG enrichment analysis of cancer stemness‐associated genes. (L) Protein‐protein interaction networks of cancer stemness‐associated genes. The thickness of line between proteins represents the interaction levels. (M) Heatmap of the correlation between stemness score and clinicopathological features and prognostic genes in gliomas (*P < .05, **P < .01, ***P < .001. ****P < .0001)

We determined the relative mRNA expression of cancer stemness‐associated genes in gliomas (Supporting information Figure S1A) and further analyzed the differential mRNA expression in normal and glioma tissues from the Oncomine database (Supporting information Figure S1B). Then, the cancer stemness‐associated genes were further analyzed by GO and KEGG enrichment analyses. The GO analyses showed that these genes were mainly involved in the regulation of cell proliferation, the activation of BMP receptors, and T‐cell activation and differentiation (Figure 1J). KEGG analyses revealed that these genes were involved in the cell cycle, cellular senescence, and immune‐related signaling pathways such as Th1 and Th2 cell differentiation signaling (Figure 1K). To evaluate the biological characteristics of the proteins encoded by the 12 genes of interest, protein‐protein interaction network analysis was performed (Figure 1L). In addition, the Pearson correlation coefficient between genes was determined, and the results were used to generate a heatmap, which indicated that the genes were highly interconnected (Supporting information Figure S5).

Gene set enrichment analysis (GSEA) is a conventional approach to identify pathways related to gene expression.^5^ The results revealed that JAK/STAT signaling, the p53 signaling pathway, and the cell cycle were significantly associated with the differentially expressed genes between the high and low stemness score groups (Supporting information Figure S2A‐C and Table S4). In addition, the expression of the 12 cancer stemness‐associated genes between the low and high stemness score groups was organized into a heatmap (Figure 1M). Next, we also observed the associations between the expression of the 12 cancer stemness‐associated genes and different WHO grades and histopathological groups (Supporting information Figures S3 and S6). These results suggested that the 12 cancer stemness‐associated genes were significantly associated with malignant behavior of gliomas.

To investigate the influence of the stemness score on prognosis, we compared the overall survival (OS) of patients in the low and high stemness score groups through the Kaplan–Meier method. Overall, the prognosis of patients in the low stemness score group was better than that of patients in the high stemness score group (hazard ratio (HR) = 0.47, 95% confidence of interval (CI) = 0.37‐0.60, *P *< .0001) (Figure 2A). To further demonstrate the prognostic value of the stemness score in glioma, we stratified the patients according to their age, sex, WHO grade, IDH status, 1p/19q status, and recurrence and reperformed survival analysis. The results showed that, consistent with the overall Kaplan–Meier curve, a high stemness score was a risk factor for poor prognosis (Supporting information Figure S7A‐L). Additionally, Supporting information Figure S4A‐B shows the distribution of stemness scores and prognoses in the 301 glioma patients.

**FIGURE 2:**
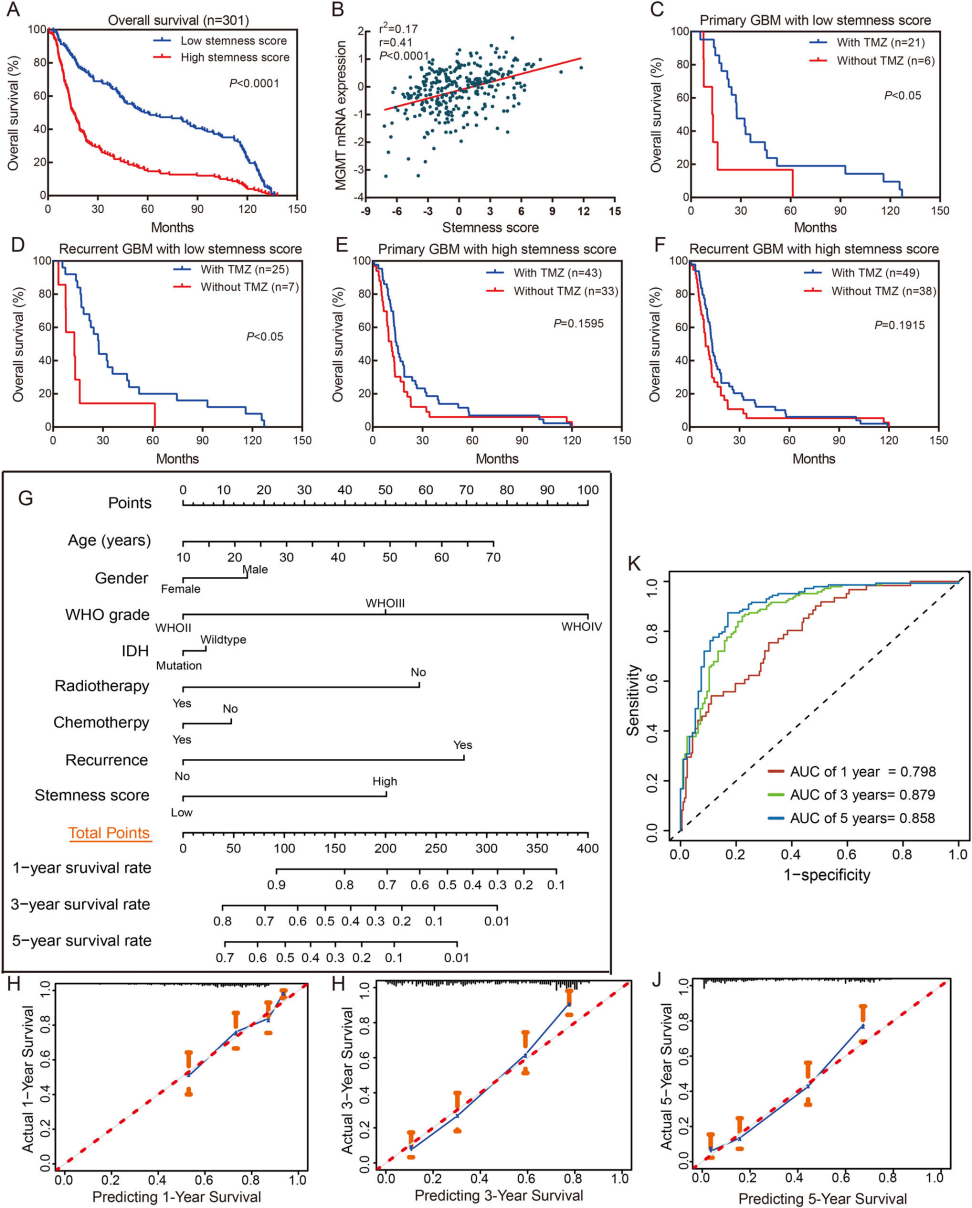
Clinical value of the stemness score in glioma patients, and construction and evaluation of Nomogram. (A) Kaplan–Meier survival curves analysis of OS between the patients with low and high stemness score in glioma. (B) Scatter plot between stemness score and MGMT mRNA expression. (C‐F) GBM patients with low stemness score had a greater benefit from chemotherapy. (G) Nomogram for predicting the 1‐, 3‐, and 5‐year survival rate for individual glioma patient. (H‐J) The calibration plots of 1‐, 3‐, and 5‐year model in glioma patients. The solid line and dotted line represent the predicted and observed values. (K) Time‐dependent ROC curve of 1‐, 3‐, and 5‐year survival model in glioma patients

To identify whether the stemness score was a novel prognostic factor for glioma patients, multivariate Cox regression analyses were performed, and the results revealed that the stemness score was a prognostic indicator for patients (HR = 2.33, 95%CI = 1.29‐4.19, *P *= .005) (Table 2).

**TABLE 2:** Univariate and multivariate Cox regression analysis of various parameters for glioma patients

	Univariate Cox regression			Multivariate Cox regression		
Characteristic	HR	95%CI	*P*‐value	HR	95%CI	*P*‐value
Age	1.47	1.16‐1.85	.001	0.80	0.44‐1.46	.459
Gender	0.72	0.57‐0.91	.006	1.36	0.79‐2.34	.274
WHO grade	1.95	1.69‐2.24	<.001	0.40	0.15‐1.12	.081
Histopathology	1.33	1.25‐1.41	<.001	2.01	1.28‐3.16	.003
IDH	0.52	0.42‐0.66	<.001	0.92	0.46‐1.83	.802
1p/19q	0.39	0.23‐0.69	.001	0.86	0.38‐1.97	.727
Radiotherapy	0.57	0.440‐0.80	.001	0.60	0.30‐1.21	.153
Chemotherapy	1.37	1.08‐1.75	.011	0.71	0.38‐1.33	.285
Recurrence	0.41	0.28‐0.59	<.001	0.51	0.27‐0.97	.039
Stemness score	2.23	1.76‐2.82	<.001	2.33	1.29‐4.19	.005

CI, confidence interval; HR, hazard ratio.

O^6^‐methylguanine DNA methyltransferase (MGMT) expression is an important molecular marker of the therapeutic effects of chemotherapeutic drugs in glioma patients.^6^ We analyzed the correlation between the stemness score and MGMT expression via a scatter plot. Interestingly, the results showed that the stemness score was positively related to MGMT expression (*r* = 0.41, *r*2 = 0.17, *P *< .0001) (Figure 2B), which suggested that patients with low stemness scores were sensitive to chemotherapy. To verify the above speculation, we compared the OS of patients treated with and without chemotherapy in the low and high stemness score groups. For patients with low stemness scores, those in the primary and recurrent GBM subgroups showed a benefit from chemotherapy (HR = 0.39, 95%CI = 0.11‐1.37, *P *< .05; HR = 0.36, 95%CI = 0.11‐1.19, *P *< .05, respectively) (Figure 2C‐D). However, for patients with high stemness scores, those in the primary and recurrent GBM subgroups did not benefit from chemotherapy (HR = 0.73, 95%CI = 0.46‐1.16, *P *= .1595; HR = 0.76, 95%CI = 0.49‐1.18, *P *= .1915, respectively) (Figure 2E‐F). Taken together, the above results suggest that a low stemness score may be a predictive marker for the response to therapy in glioma patients. However, whether the identified genes are key genes responsible for chemotherapy resistance in glioma patients needs to be further confirmed by in vivo and in vitro experiments.

A nomogram was constructed by integrating the stemness score and clinical features (Figure 2G). The total point for the nomogram consisted of the sum of the patient age, sex, WHO grade, IDH, radiotherapy, chemotherapy, recurrence and stemness scores, and the higher total point was correlated with worse prognosis. To assess the predictive effects of the nomogram, Kaplan–Meier curves, calibration curves, and time‐dependent ROCs were drawn. The Kaplan–Meier curves revealed that the OS of patients in the low total points group was significantly better than that of patients in the high total points group (HR = 0.30, 95%CI = 0.23‐0.40, *P *< .0001), and the median OS of the two groups was 94.3 and 13.8 months, respectively (Supporting information Figure S8). The calibration curve showed good agreement between the predicted probability of survival and actual of survival (Figure 2H‐J). According to the results of the time‐dependent ROC analysis, the area under curve (AUCs) for the 1‐, 3‐, and 5‐year survival rates of glioma patients were0.798, 0.897, and 0.858, respectively, which suggested that the nomogram had good performance in prognosis prediction (Figure 2K). Furthermore, we also used another dataset of 325 patients (ID: mRNAseq_325, Supporting information Table S2) of CGGA as a validation cohort to verify the final model. The time‐dependent ROC curve and calibration curves for 1‐, 3‐, and 5‐year survival were generated, as shown in Supporting information Figure S9. The AUCs for the 1‐, 3‐, and 5‐year survival of glioma patients were 0.792, 0.845, and 0.839, respectively. There was excellent agreement between the results obtained from the discovery and validation cohorts.

In our research, we identified a 12‐gene prognostic signature for patients with glioma. A stemness score based on the 12 genes was associated with clinicopathological features and a novel prognostic factor in gliomas. Additionally, a low stemness score may serve as an indicator of the response to chemotherapy. More importantly, a nomogram including clinical features and the stemness score was constructed and was able to precisely predict the prognosis of patients, suggesting its utility in the individualized treatment of glioma patients.

## CONFLICT OF INTEREST

The authors declare no conflict of interest.

## Supporting information

Supporting information
