# Supplementary material for: Prognostic significance of cancer stemness‐associated genes in patients with gliomas
Source: Clin Transl Med. 2020 Sep 27;10(5):e186. doi: 10.1002/ctm2.186 (PMC7520082; doi:10.1002/ctm2.186)
Supplement: Supplementary file 1 — Supporting information [file CTM2-10-e186-s001.pdf]

# Supplementary information

## This PDF file includes:

## Catalogue

MATERIALS AND METHODS ........ 2
Data Collection ........ 2
Selection of Cancer Stemness-associated Genes ........ 2
Screening of Prognostic Cancer Stemness-associated Genes ........ 2
Biological Function Analysis ........ 3
Correlation of Prognostic Genes and Interaction network of Proteins ........ 4
Construction and Validation of Nomogram ........ 4
Statistical Analysis ........ 4
Figure S1. ........ 5
Figure S2. ........ 6
Figure S3. ........ 7
Figure S4. ........ 8
Figure S5. ........ 9
Figure S6. ........ 10
Figure S7. ........ 11
Figure S8. ........ 12
Figure S9 ........ 13
Table S1. ........ 14
Table S2. ........ 15
Table S3. ........ 16
Table S4. ........ 17
REFERENCES ........ 18

## MATERIALS AND METHODS

### Data Collection

The mRNA microarray data of 301 patients (ID: mRNA-array, Data Type: mRNA Microarray) with gliomas were extracted from Chinese Glioma Genome Atlas (CGGA) database (http://cgga.org.cn/download.jsp) [1]. All transcriptome data of patients were transformed by Log2. The clinical features and follow-up data were extracted by two doctors.

### Selection of Cancer Stemness-associated Genes

Firstly, we searched for the all genes associated with the word of “Cancer stem cell” in comprehensive database of human genes, namely, GeneCards database (https://www.genecards.org/). Secondly, the genes retrieved were further screened by the relevance score ranging from 0 to 100 points, which indicated the correlation between the genes and cancer stem cell. The higher the score, the more relevant. We screened 359 genes with relevance score ≥50 points as the object of further study.

### Screening of Prognostic Cancer Stemness-associated Genes

The genes screened in a previous step were included into univariate Cox analysis. Then, the genes with *P*-value≤0.001 were selected from the univariate analysis results and further

incorporated into multivariate Cox analysis. The genes with *P*-value≤0.001 considered as the independent prognostic genes for glioma patients in the results of multivariate analysis.

Next, stemness-score of each patient was calculated by gene mRNA levels multiplied by the regression regression coefficient ($\beta$). The formula of stemness-score was listed as follows: Stemness-score=$gene_1 * \beta_1 + gene_2 * \beta_2 + gene_3 * \beta_3$ ……. $+gene_{(n)} * \beta_{(n)}$. All patients were divided into low and high stemness-score group based on the median cut-off of stemness-score. $\chi^2$ was performed to analyze the correlation between stemness-score and clinical features.

**Biological Function Analysis**

To investigate the biological function of prognostic genes, we retrieved the top 10 genes which were significantly co-expressed with each prognostic gene from the Cbioportal database (http://www.cbioportal.org/) [2]. The functional annotation of Gene Ontology (GO) was conducted by the cytoscape tool (https://cytoscape.org/) [3]. The same tool was also used to implement the Kyoto Encyclopedia of Genes and Genomes (KEGG) analysis. In addition, we also performed Gene Set Enrichment Analysis (GSEA) to identify the signaling pathways in which differential expression genes between high and low stemness-score groups were enriched (http://software.broadinstitute.org/gsea/) [4]. The significant values were as follows: *P*<0.01, the absolute value of normalized enrichment score (NES)>1 and the false discovery rate (FDR) ≤0.25.

## Correlation of Prognostic Genes and Interaction network of Proteins

Next, the String (https://string-db.org/) [5] database was used to observe the protein interaction network between proteins encoded by prognostic genes. Additionally, the R “corrplot” package was used to calculate the Pearson correlation coefficient between genes.

## Construction and Validation of Nomogram

Clinical factors and stemness-score were both used to construct a prediction model by using “Survival” package and “rms” package. Next, calibration and time-dependent ROC curves were plotted to assess the accuracy of the nomogram. The Kaplan-Meier curve was also performed to evaluate the nomogram.

## Statistical Analysis

All statistics were performed by SPSS 23.0 or R software 3.6.5. The difference of classified data was detected by $\chi^2$ test. The difference of the OS between two groups was compared by log-rank test in Kaplan-Meier curves analysis. The area under the ROC curve of the time-dependent ROC curve served as an indicator of prediction accuracy. In this study, *P*-value <0.05 (two-sided test) were considered to be statistically significant. Bonferroni correction was applied in multiple group comparisons.

Figure S1.

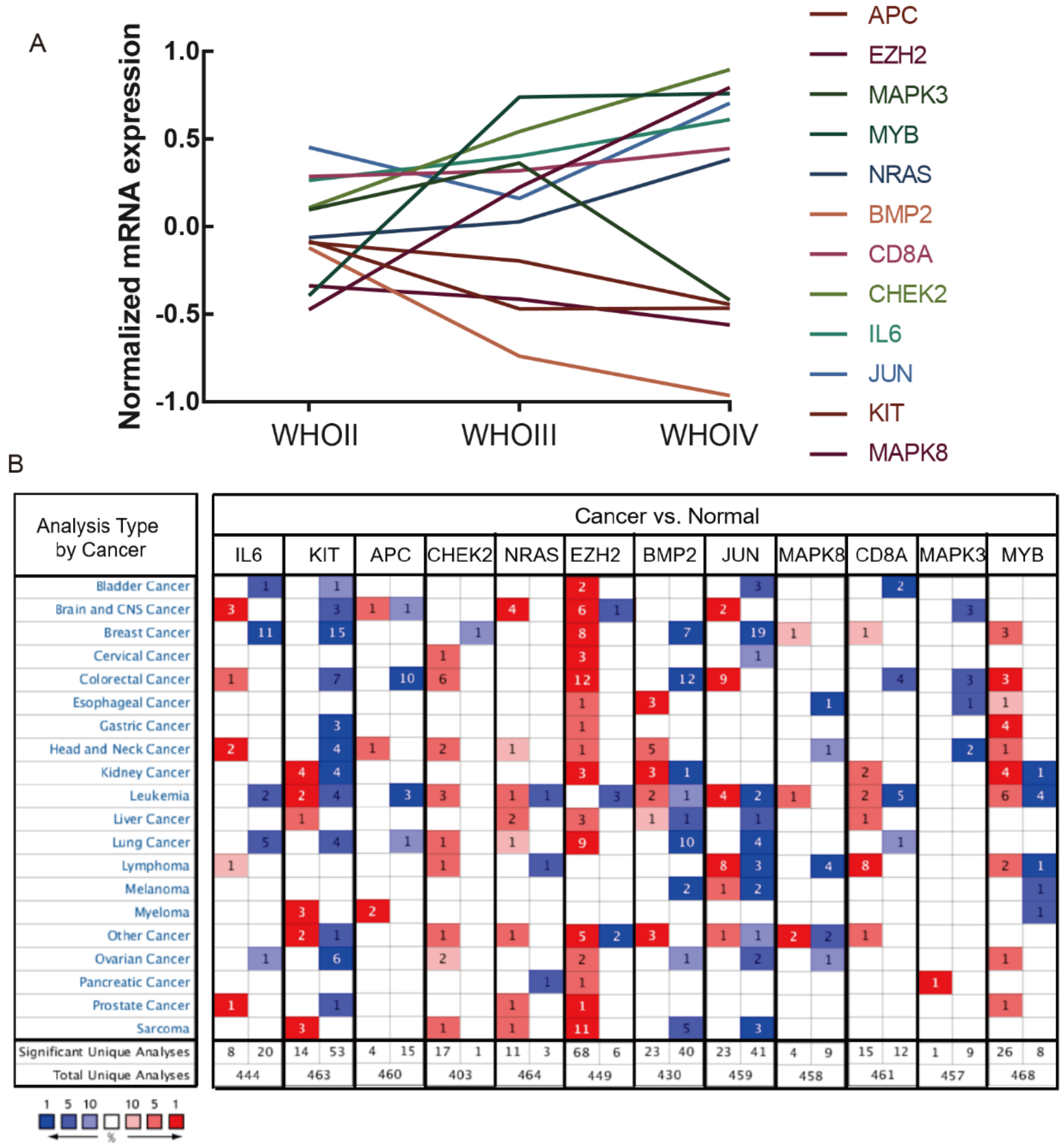


**Figure S1.** Relative mRNA expression of Cancer Stemness-associated genes. (A) Trend of relative mRNA expression of Cancer Stemness-associated genes in gliomas. (B) The mRNA expression patterns of cancer stemness-associated genes between tumors and normal tissue in overall cancers from Oncomine database. The red indicates high expression, and the blue indicates low expression.

Figure S2.

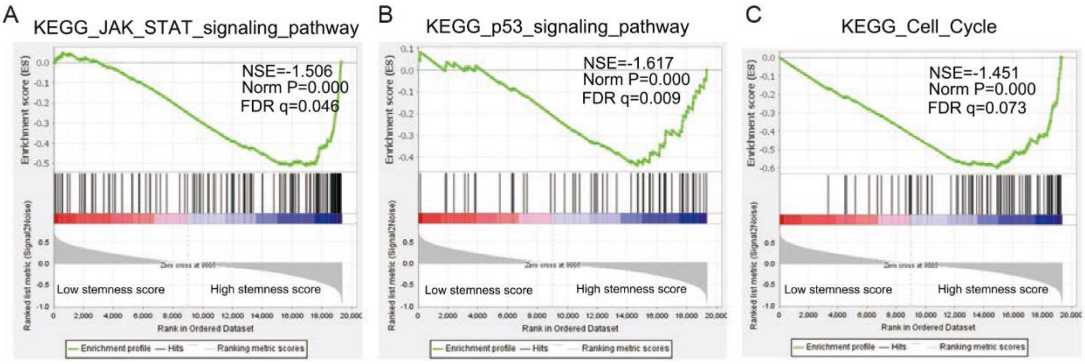


**Figure S2.** GSEA analysis showed that high stemness score was positively correlated with three cancer pathways: (A) JAK/STAT signaling pathway, (B) p53 signaling pathway and (C) Cell Cycle.

Figure S3.

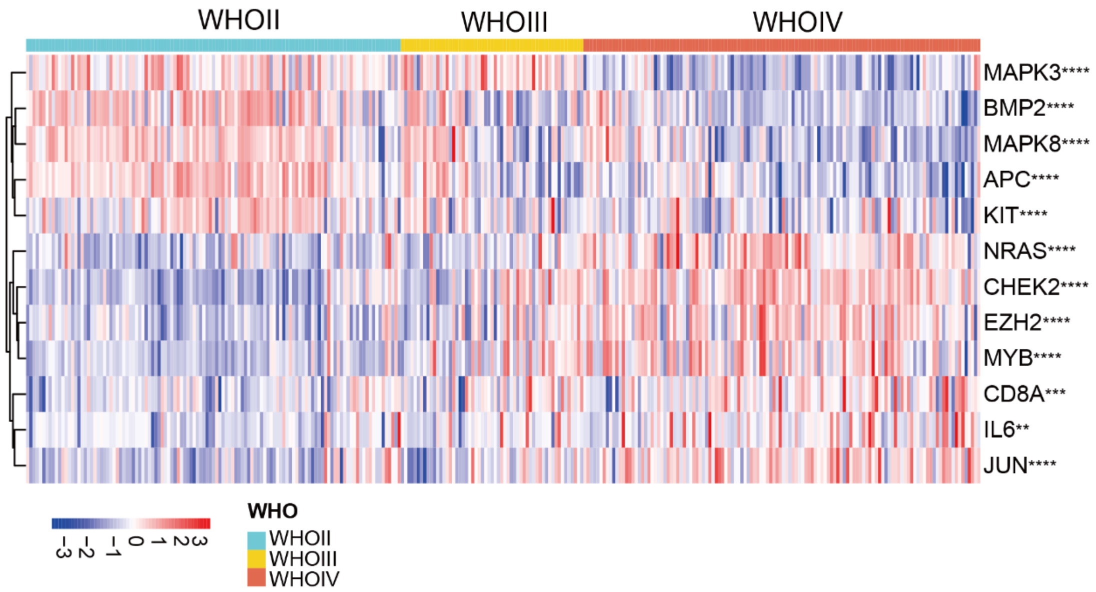


**Figure S3.** Heatmap of the correlation between different WHO grade and prognostic genes in gliomas (**P*<0.05, ***P*<0.01, ****P*<0.001. *****P*<0.0001).

Figure S4.

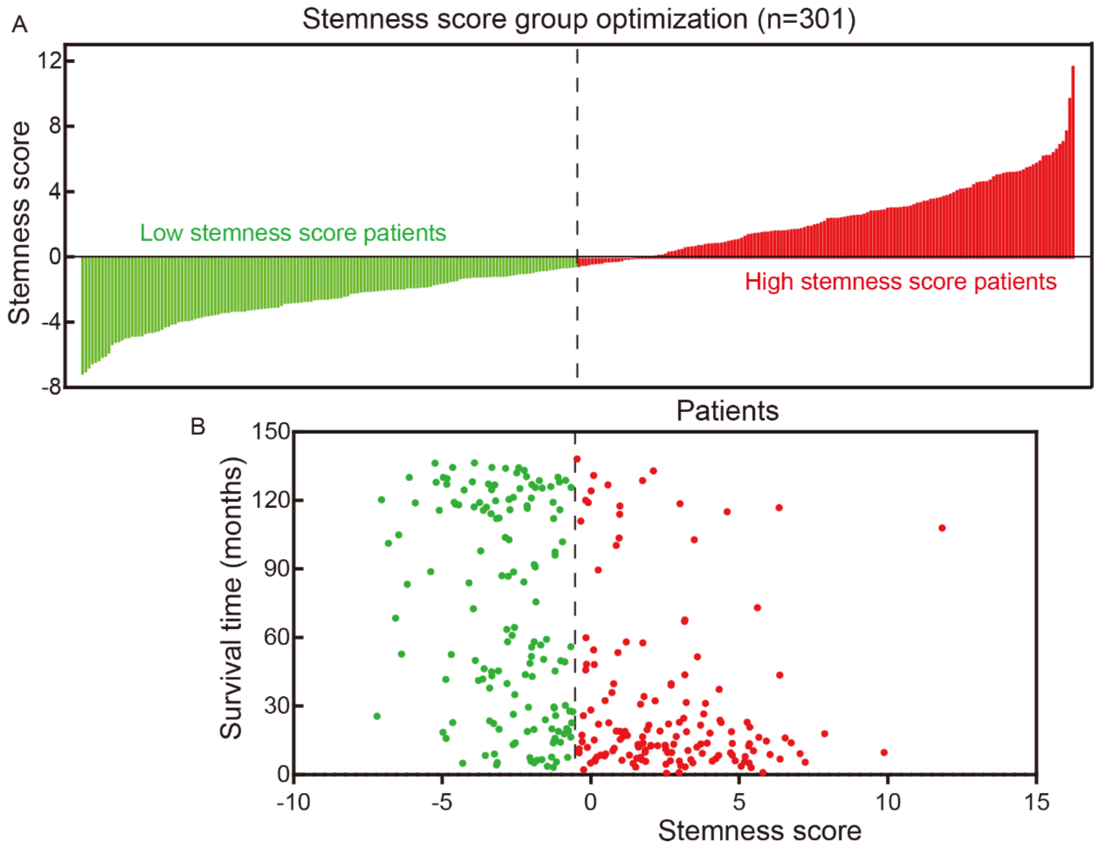


**Figure S4.** Correlation between stemness score and survival time in 301 patients. (A) The distributions of stemness score in 301 glioma patients. (B) The correlation between survival time and stemness score of each patient. Green represents low stemness score, and red represents high stemness score.

Figure S5.

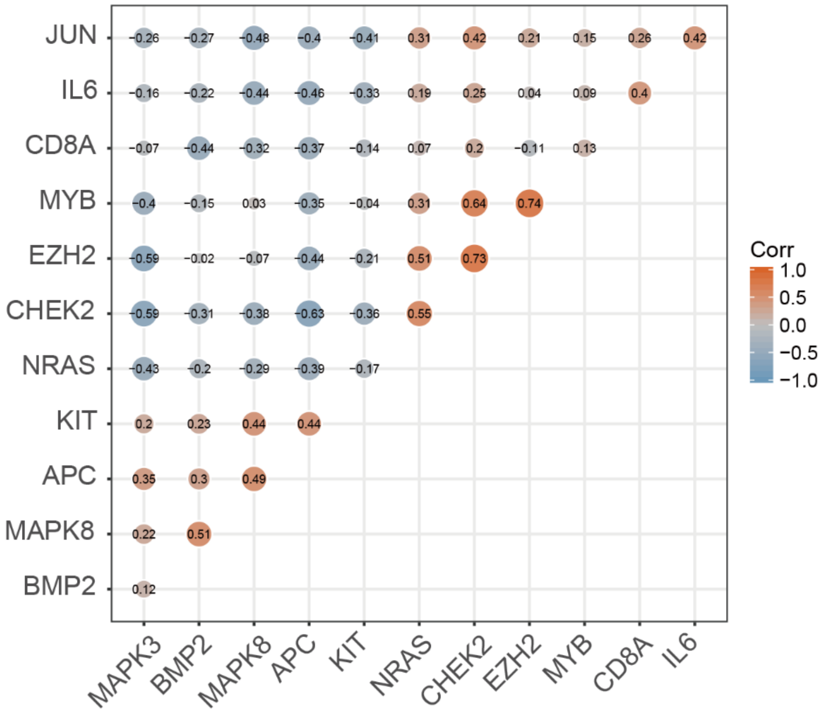


**Figure S5.** Correlation matrix between cancer stemness-associated genes in glioma patients.

The values of heatmap represent Pearson correlation coefficients.

Figure S6.

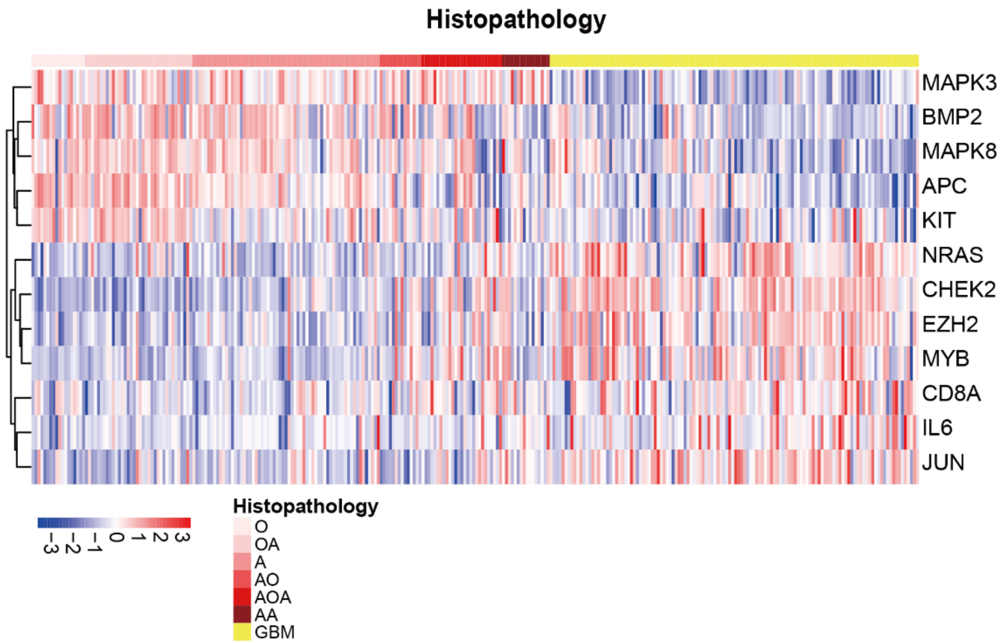


**Figure S6.** Heatmap of the correlation between different histopathology and prognostic genes in gliomas.

Figure S7.

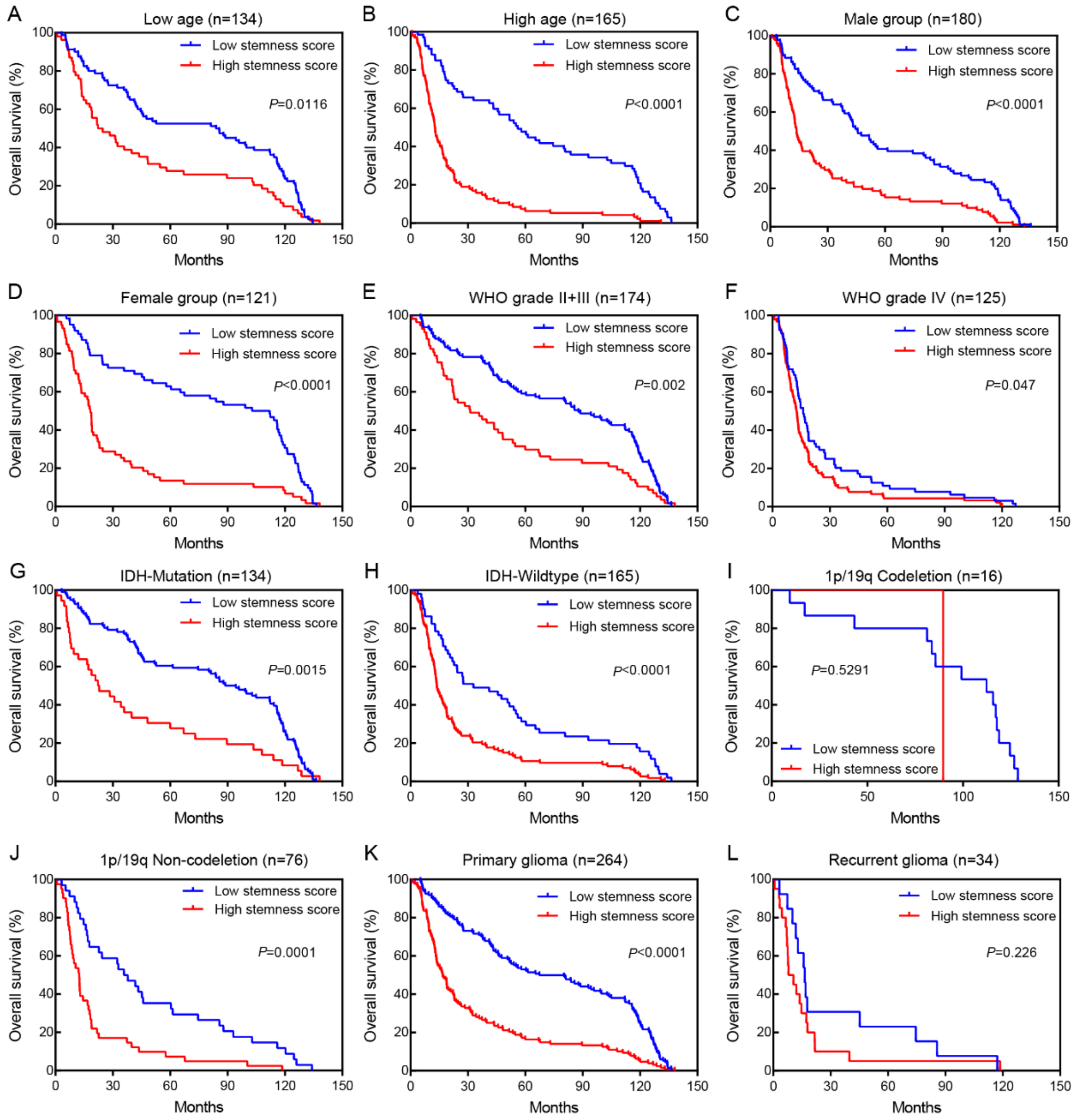


**Figure S7.** Kaplan-Meier curve analysis of stemness score in different subgroups. Prognostic value of the stemness score in patients stratified by age, gender, WHO grade, IDH status, 1p/19q status and tumor recurrence. Kaplan-Meier overall survival curves for patients in low age subgroup (A), high age subgroup (B), male subgroup (C), female subgroup (D), WHO grade II+III subgroup (E), WHO grade IV subgroup (F), IDH mutation subgroup (G), IDH wildtype subgroup (H), 1p/19q codeletion subgroup (I), 1p/19q non-codeletion subgroup (J),

primary tumor subgroup (K) and recurrent tumor subgroup (L).

Figure S8.

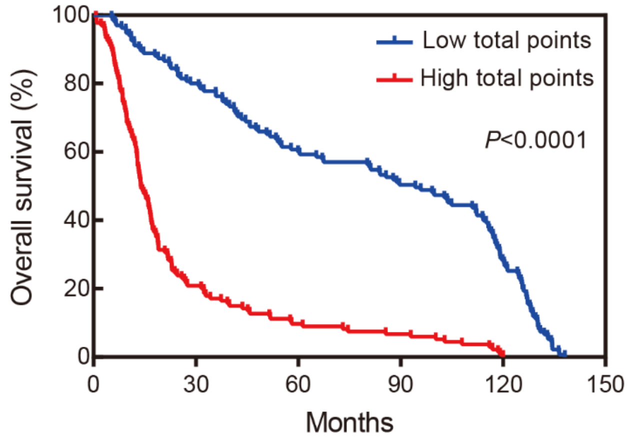


**Figure S8.** Kaplan-Meier survival curves analysis of OS between low and high total points groups in glioma patients.

**Figure S9**

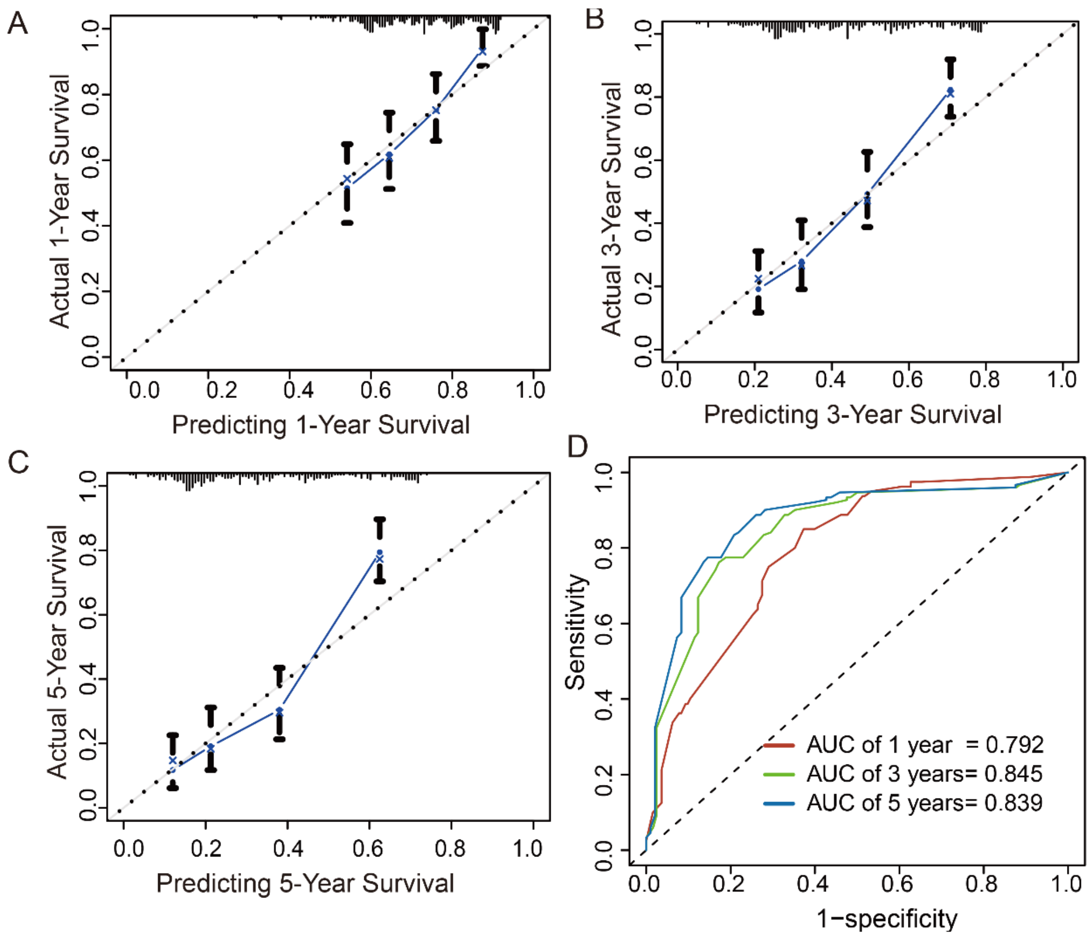


**Figure S9.** Verification of the prediction model in validation cohort of glioma patients. (A-C) The calibration curves of 1-, 3- and 5-year model in validation cohort. (D) Time-dependent ROC curve of 1-, 3- and 5-year survival model in validation cohort.

Table S1.

**Table S1.** Univariate and multivariate Cox regression analysis of cancer stemness-associated genes for glioma patients.

| Signature | Univariate Cox regression | | | Multivariate Cox regression | | |
|---|---|---|---|---|---|---|
| | **HR** | **95%CI** | ***P*-value** | **HR** | **95%CI** | ***P*-value** |
| APC | 0.47 | 0.39-0.58 | <0.001 | 0.14 | 0.05-0.4 | <0.001 |
| EZH2 | 1.42 | 1.28-1.58 | <0.001 | 4.35 | 2.34-8.06 | <0.001 |
| MAPK3 | 0.55 | 0.45-0.67 | <0.001 | 6.55 | 2.53-16.91 | <0.001 |
| MYB | 1.4 | 1.27-1.55 | <0.001 | 0.32 | 0.2-0.51 | <0.001 |
| NRAS | 1.68 | 1.4-2 | <0.001 | 0.27 | 0.15-0.48 | <0.001 |
| BMP2 | 0.79 | 0.74-0.84 | <0.001 | 0.58 | 0.42-0.8 | 0.001 |
| CD8A | 1.33 | 1.17-1.51 | <0.001 | 2.44 | 1.43-4.16 | 0.001 |
| CHEK2 | 1.63 | 1.45-1.84 | <0.001 | 2.85 | 1.53-5.32 | 0.001 |
| IL6 | 1.21 | 1.11-1.32 | <0.001 | 0.54 | 0.38-0.76 | 0.001 |
| JUN | 1.42 | 1.26-1.61 | <0.001 | 2.16 | 1.35-3.48 | 0.001 |
| KIT | 0.81 | 0.73-0.90 | <0.001 | 1.70 | 1.25-2.3 | 0.001 |
| MAPK8 | 0.53 | 0.44-0.65 | <0.001 | 0.16 | 0.06-0.47 | 0.001 |

HR: hazard ratio; CI: confidence interval.

**Table S2.**

**Table S2.** Characteristics of patients in discovery cohort and validation cohort.

| | | Discovery Cohort | | Validation Cohort | |
|---|---|---|---|---|---|
| **Characteristics** | **Category** | **No. of cases** | **(%)** | **No. of cases** | **(%)** |
| **Age(years)** | <40 | 134 | 44.5 | 130 | 40 |
| | ≥40 | 165 | 54.8 | 195 | 60 |
| | NA | 2 | 0.7 | 0 | 0.0 |
| **Gender** | Male | 180 | 59.8 | 203 | 62.5 |
| | Female | 121 | 40.2 | 122 | 37.5 |
| **WHO grade** | WHOII | 117 | 38.9 | 103 | 31.7 |
| | WHOIII | 57 | 18.9 | 79 | 24.3 |
| | WHOIV | 124 | 41.2 | 139 | 42.8 |
| | NA | 3 | 1.0 | 4 | 1.2 |
| **Histopathology** | O | 18 | 6.0 | 26 | 8.0 |
| | OA | 36 | 12.0 | 38 | 11.7 |
| | A | 63 | 20.9 | 39 | 12.0 |
| | AO | 14 | 4.6 | 12 | 3.7 |
| | AOA | 27 | 9.0 | 39 | 12.0 |
| | AA | 16 | 5.3 | 28 | 8.6 |
| | GBM | 124 | 41.2 | 139 | 42.8 |
| | NA | 3 | 1.0 | 4 | 1.2 |
| **IDH** | Mutation | 134 | 44.5 | 175 | 53.8 |
| | Wildtype | 165 | 54.8 | 46.0 | 45.8 |
| | NA | 2 | 0.7 | 1 | 0.3 |
| **1p/19q** | Codel | 16 | 5.3 | 67 | 20.6 |
| | Non-codel | 76 | 25.2 | 250 | 76.9 |
| | NA | 209 | 69.5 | 8 | 2.5 |
| **Radiotherapy** | Yes | 249 | 82.7 | 258 | 79.4 |
| | No | 38 | 12.6 | 51 | 15.7 |
| | NA | 14 | 4.7 | 16 | 4.9 |
| **Chemotherapy** | Yes | 151 | 50.1 | 178 | 54.8 |
| | No | 126 | 41.9 | 124 | 38.2 |
| | NA | 24 | 8.0 | 23 | 7.1 |
| **Recurrence** | Yes | 34 | 11.3 | 61 | 18.8 |
| | No | 264 | 87.7 | 248 | 76.3 |
| | NA | 3 | 1.0 | 16 | 4.9 |

O: oligodendroglioma; OA: oligoastrocytoma; A: astrocytoma; AO: anaplastic oligodendroglioma; AOA: anaplastic oligoastrocytoma; AA: anaplastic astrocytoma; GBM: glioblastoma; NA: missing value.

**Table S3.**

**Table S3.** The characteristics and regression coefficient of 12 cancer stemness-associated genes.

| Gene | Description | Category | Coefficient | Relevance score |
|---|---|---|---|---|
| IL6 | Interleukin 6 | Protein Coding | -0.62 | 160.09 |
| KIT | KIT Proto-Oncogene, Receptor Tyrosine Kinase | Protein Coding | 0.53 | 145.25 |
| APC | APC Regulator Of WNT Signaling Pathway | Protein Coding | -1.97 | 127.82 |
| CHEK2 | Checkpoint Kinase 2 | Protein Coding | 1.05 | 121.05 |
| NRAS | NRAS Proto-Oncogene, GTPase | Protein Coding | -1.31 | 101.88 |
| EZH2 | Enhancer Of Zeste 2 Polycomb Repressive Complex 2 Subunit | Protein Coding | 1.47 | 94.19 |
| BMP2 | Bone Morphogenetic Protein 2 | Protein Coding | -0.54 | 86.87 |
| JUN | Jun Proto-Oncogene, AP-1 Transcription Factor Subunit | Protein Coding | 0.77 | 79.15 |
| MAPK8 | Mitogen-Activated Protein Kinase 8 | Protein Coding | -1.83 | 74.37 |
| CD8A | CD8a Molecule | Protein Coding | 0.89 | 69.7 |
| MAPK3 | Mitogen-Activated Protein Kinase 3 | Protein Coding | 1.88 | 68.28 |
| MYB | MYB Proto-Oncogene, Transcription Factor | Protein Coding | -1.14 | 51.08 |

**Table S4.**

**Table S4.** Top ranked terms in GSEA analysis results for glioma patients with high stemness scores.

| **GSEA Term Name** | **ES** | **NES** | **NOM *p*-val** | **FDR q-val** |
|---|---|---|---|---|
| Toll Like Receptor Signaling Pathway | -0.446 | -1.868 | <0.001 | <0.001 |
| Jak Stat Signaling Pathway | -0.394 | -1.506 | <0.001 | 0.046 |
| B Cell Receptor Signaling Pathway | -0.370 | -1.844 | <0.001 | <0.001 |
| P53 Signaling Pathway | -0.541 | -1.617 | <0.001 | 0.009 |
| Cell Cycle | -0.479 | -1.451 | <0.001 | 0.073 |
| T Cell Receptor Signaling Pathway | -0.340 | -1.371 | <0.001 | 0.088 |
| DNA Replication | -0.644 | -1.479 | <0.001 | 0.068 |
| Focal Adhesion | -0.470 | -1.333 | <0.001 | 0.128 |
| Adherens Junction | -0.315 | -1.297 | <0.001 | 0.151 |
| Cytokine Cytokine Receptor Interaction | -0.503 | -2.196 | <0.001 | <0.001 |
| Drug Metabolism Other Enzymes | -0.483 | -1.689 | <0.001 | <0.001 |
| Regulation of Actin Cytoskeleton | -0.367 | -1.667 | <0.001 | 0.010 |

ES: Enrichment Score; NES: Normalized Enrichment Score; FDR: False Discovery Rate.

# REFERENCES


1. Fang S, Liang J, Qian T*, et al.* Anatomic Location of Tumor Predicts the Accuracy of Motor Function Localization in Diffuse Lower-Grade Gliomas Involving the Hand Knob Area. *AJNR Am J Neuroradiol* 2017; 38: 1990-1997.
2. Cerami E, Gao J, Dogrusoz U*, et al.* The cBio cancer genomics portal: an open platform for exploring multidimensional cancer genomics data. *Cancer Discov* 2012; 2: 401-404.
3. Shannon P, Markiel A, Ozier O*, et al.* Cytoscape: a software environment for integrated models of biomolecular interaction networks. *Genome Res* 2003; 13: 2498-2504.
4. Subramanian A, Tamayo P, Mootha VK*, et al.* Gene set enrichment analysis: a knowledge-based approach for interpreting genome-wide expression profiles. *Proc Natl Acad Sci U S A* 2005; 102: 15545-15550.
5. Szklarczyk D, Gable AL, Lyon D*, et al.* STRING v11: protein-protein association networks with increased coverage, supporting functional discovery in genome-wide experimental datasets. *Nucleic Acids Res* 2019; 47: D607-d613.
